# Demonstration of polarization control GaN-based micro-cavity lasers using a rigid high-contrast grating reflector

**DOI:** 10.1038/s41598-019-49604-0

**Published:** 2019-09-10

**Authors:** Tsu-Chi Chang, Kuo-Bin Hong, Shuo-Yi Kuo, Tien-Chang Lu

**Affiliations:** Department of Photonics, College of Electrical and Computer Engineering, National Chiao Tung University, Hsinchu, 30010 Taiwan

**Keywords:** Semiconductor lasers, Microresonators

## Abstract

We reported on GaN microcavity (MC) lasers combined with one rigid TiO_2_ high-contrast grating (HCG) structure as the output mirror. The HCG structure was directly fabricated on the GaN structure without an airgap. The entire MC structure comprised a bottom dielectric distributed Bragg reflector; a GaN cavity; and a top HCG reflector, which was designed to yield high reflectance for transverse magnetic (TM)- or transverse electric (TE)-polarized light. The MC device revealed an operation threshold of approximately 0.79 MW/cm^2^ when pulsed optical pumping was conducted using the HCG structure at room temperature. The laser emission was TM polarized with a degree of polarization of 99.2% and had a small divergence angle of 14° (full width at half maximum). This laser operation demonstration for the GaN-based MC structure employing an HCG exhibited the advantages of HCGs in semiconductor lasers at wavelengths from green to ultraviolet.

## Introduction

Around the world, GaN-based microcavity (MC) structures used for resonant cavity light-emitting diodes, edge-emitting lasers, vertical cavity surface-emitting lasers (VCSELs), and polaritonic emitters have recently received considerable attention^1–7^. Distributed Bragg reflectors (DBRs) are commonly used to create high quality factor MCs for these devices to realize simple but robust structures. However, recently, HCGs have been investigated for use as reflectors in microcavities and lasers^8,9^. The HCG structure comprises a single layer of a periodic subwavelength grating that comprises a high-refractive-index material surrounded by a low-refractive-index material. An HCG has been demonstrated to have a number of superior characteristics such as a relatively wide stopband, adequate reduction in the thickness of the mirror, and polarization-dependent feedback. Moreover, when an HCG is used, the resonance wavelength can be set based on the grating parameters, which enables the fabrication of a multiwavelength VCSEL array from a single epitaxial structure^10^. By using an HCG, rapidly tunable VCSELs can be fabricated by modulating the vertical position of HCG membranes^10,11^. To date, electrically driven VCSELs with HCG reflectors in the infrared spectral regime have been reported and widely studied^8–14^. In many cases, the top mirror comprises a thin membrane HCG layer combined with a few *λ*/4-thick DBR layer pairs to boost the reflectance. There have been a few studies in which the top DBR has been completely replaced by a thin membrane HCG^11–16^. Nevertheless, lasing was still achieved under electrical injection. The HCG layer is usually surrounded by air to achieve the largest refractive index contrast between the grating and the surrounding material. This provides a high reflectance value from the grating that is not strongly dependent on the grating parameters. Such a value in turn yields a large fabrication tolerance window.

GaN-based emitters with HCG structures have not yet been widely investigated to an equivalent level due to the difficulty in creating a free-standing grating surrounded by air with a low refractive index. The reason for the difficulty is that the III-nitride material system has no appropriate sacrificial material that can be selectively etched without destructing the HCG structure. In the III-nitride material system, HCGs have been realized using photo-electro-chemical etching or focused ion beam (FIB) milling^17–22^ for producing an airgap embedded inside a device. The photo-electro-chemical method entails a risk of damaging the multiple quantum well structure. The use of the focused ion beam method imposes a limitation on the airgap height. Due to the difficulties observed in realizing a III-nitride-based HCG structure with an airgap and in achieving a structure that has high strength and stiffness, a GaN grating reflector without an airgap has been proposed^18,23^. Fabricating an HCG structure for use in devices with blue and UV wavelengths is challenging because the fabrication window of an HCG structure with a high reflectance value is excessively narrow. In our study, a TiO_2_ HCG structure was grown directly on a GaN surface. This design provides a refractive index contrast that is analogous to that of an HCG in a GaN. As lattice matching between the grating and the MC structure is not required, this dielectric HCG structure can be implemented on an MC in many different material systems and thus be used for many different wavelength regimes. In this study, we demonstrated the lasing action in a GaN MC with an HCG reflector under pulsed optical pumping at room temperature for the first time.

## Experiment

**Figure 1:**
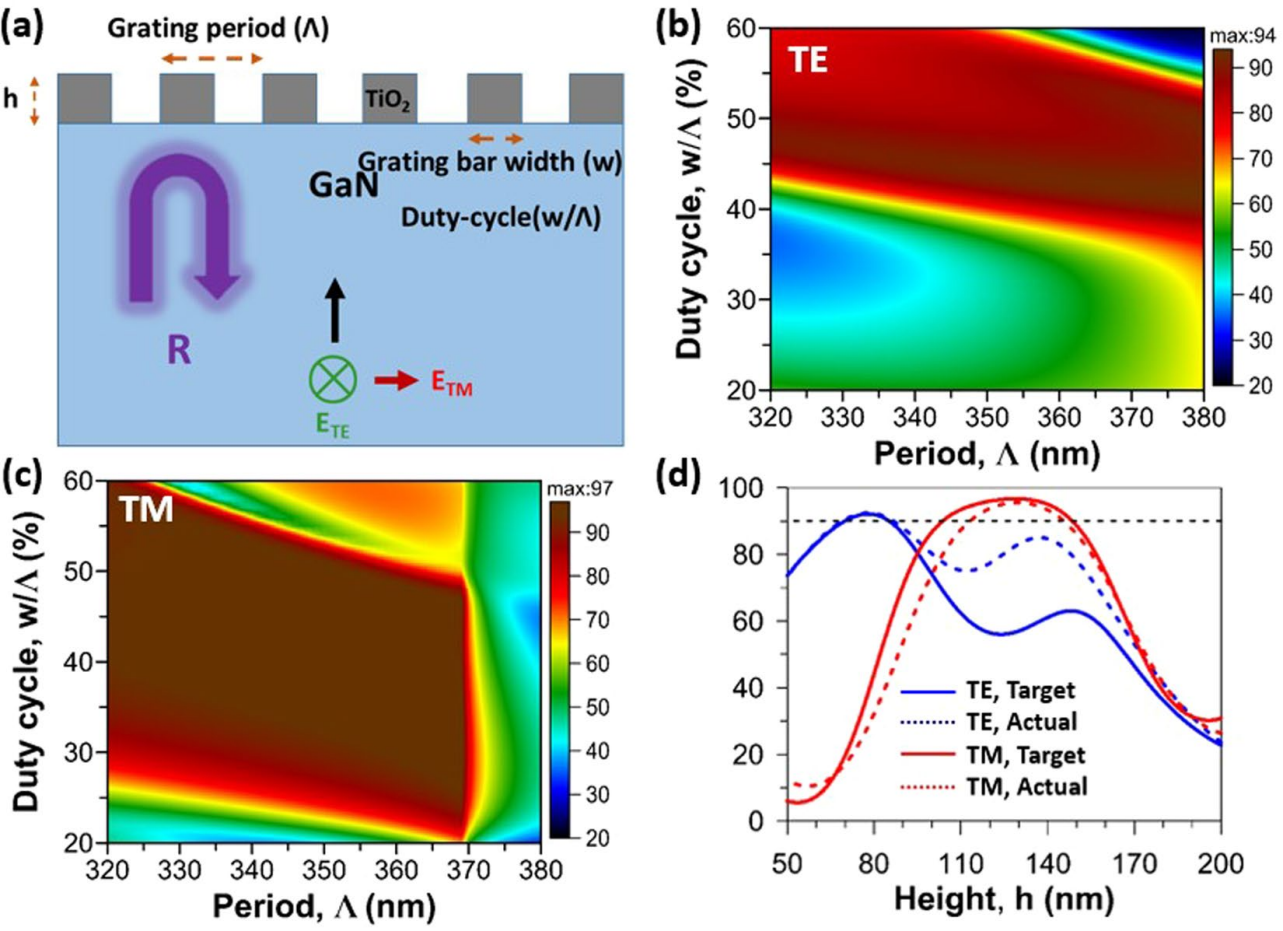
(**a**) Sketch drawing of a TiO_2_ HCG reflector on GaN film for structural design. The reflectance maps as a function of DC and period for (**b**) TE and (**c**) TM polarized light with a wavelength of 369.3 nm. A grating height of 75 nm has been used for an HCG for TE-polarized light and 130 nm for an HCG for TM-polarized light. (**d**) Simulated reflectance versus the grating height for TM (red lines) and TE (blue lines) polarizations. The solid lines indicate the target structure containing a 368 nm period grating with 32% DC and dashed lines express the calculated reflectance for the actual HCG grating with period of 344 nm and DC of 42% extracted from the SEM image.

The schematic of a TiO_2_-based HCG fabricated on a GaN film for use as a structural design is shown in Fig. 1(a). The figure includes the definitions of the design parameters—the grating height *h*, grating period *Λ*, grating bar width *w*, and duty cycle (DC) *w*/*Λ*. For achieving a high reflectance value at a specific wavelength that coincided with the gain peak of GaN, the plane waves with transverse magnetic (TM) and transverse electric (TE) polarizations were normally incident on the GaN facet of the TiO_2_ grating on the GaN film; scattering parameters (S-parameters) were numerically calculated by employing COSMOL Multiphysics finite-element analysis software. To properly design and optimize a TiO_2_-based HCG mirror with high reflectance, the complex refractive index of TiO_2_ should be considered in the calculation due to the non-negligible optical absorption (large extinction coefficient of TiO_2_) in the ultraviolet regime^24^. Fig. 1(b,c) illustrate the calculated reflectance maps versus the period and DC of the HCG grating under TE- and TM-polarized illumination at a wavelength of 369.3 nm, respectively. The tolerance windows for fabrication imperfections for the DC, for the period, and for the grating height are larger for a grating designed for TM-polarized light compared with the windows for a grating designed for TE-polarized light. As an example, to achieve a reflectance value greater than 90%, the grating height should be between 115 and 145 nm for TM-polarized light and must be between 69 and 86 nm for TE-polarized light, as shown in Fig. 1(d). In addition to a larger fabrication tolerance window, an HCG designed for TM-polarized light yields a higher peak reflectance value. Thus, a TM-polarized design was selected for the HCG with the target to achieve a grating height of 130 nm, DC of 32%, and period of 368 nm. Figure 1(d) displays the TE and TM reflectance spectra for target and actual HCG structures (a grating with a period of 344 nm and a DC of 42% extracted from the scanning electron microscope (SEM) image) obtained by simulation. Even when the actual HCG parameters deviated from the target design, the TM reflectance spectra presented high reflectivity for grating heights between 125 and 145 nm.

## Results and Discussion

**Figure 2:**
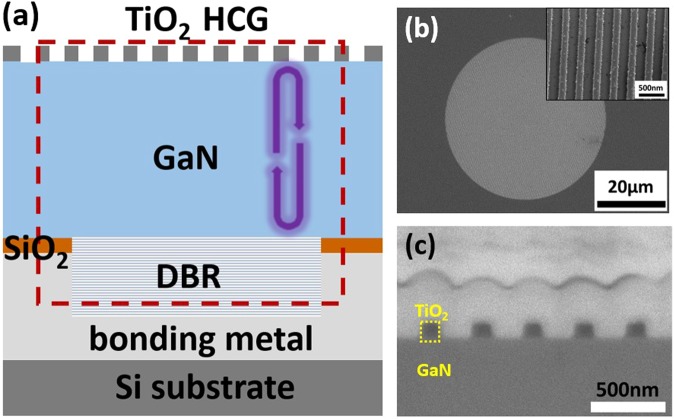
(**a**) Schematic cross-section of the GaN-based HCG MC laser consisting of a bottom SiO_2_/Ta_2_O_5_ DBR, a GaN cavity, and an HCG reflector. The red dashed square and blue curved-arrow indicate the simulation domain for calculating the output emission intensity spectrum and the reciprocating longitudinal wave. (**b**) A plane-view SEM image of the fabricated TiO_2_ HCG MC with a diameter of 45 µm. Inset shows the magnified HCG morphology. (**c**) A cross-sectional SEM image of the TiO_2_ HCG.

A schematic of the TiO_2_-based HCG MC laser is presented in Fig. 2. The epitaxial structure comprises a GaN layer grown on a c-plane patterned sapphire substrate. First, a thin nucleation layer of GaN followed by a bulk GaN layer was grown. Second, after the epitaxial growth, a dielectric DBR (12 pairs of SiO_2_/Ta_2_O_5_) was deposited using an E-gun evaporator. Third, flip-chip metal bonding to a Si substrate was conducted. Fourth, the laser-induced lift-off process was used to remove the sapphire substrate. Fifth, the GaN was polished to a thickness of approximately 5.15 µm, and the surface roughness after polishing was 0.673 nm (root-mean-square determined by atomic force microscopy over a scan area of 5 × 5 µm^2^). Sixth, the TiO_2_-grating layer was deposited, followed by a SiO_2_ layer. The SiO_2_ layer was used as a hard mask together with the Ni mask patterned by e-beam lithography through the lift-off process. Finally, the grating was dry etched and the hard mask was removed. Thus, a TiO_2_ HCG was directly grown on the GaN surface, as shown in the SEM image in Fig. 2(b). To examine the fine parameters of the HCG, FIB etching was used to reveal the cross-section of the TiO_2_ HCG. The resulting structure is presented in Fig. 2(c), including the platinum layer deposited on top of the grating for protection during FIB etching. The TiO_2_ height was approximately 120–130 nm, the period was nearly 340–350 nm, and the DC was 42%. All these parameter values are near the targeted design parameter values for a high reflectance mirror for TM-polarized light.

**Figure 3:**
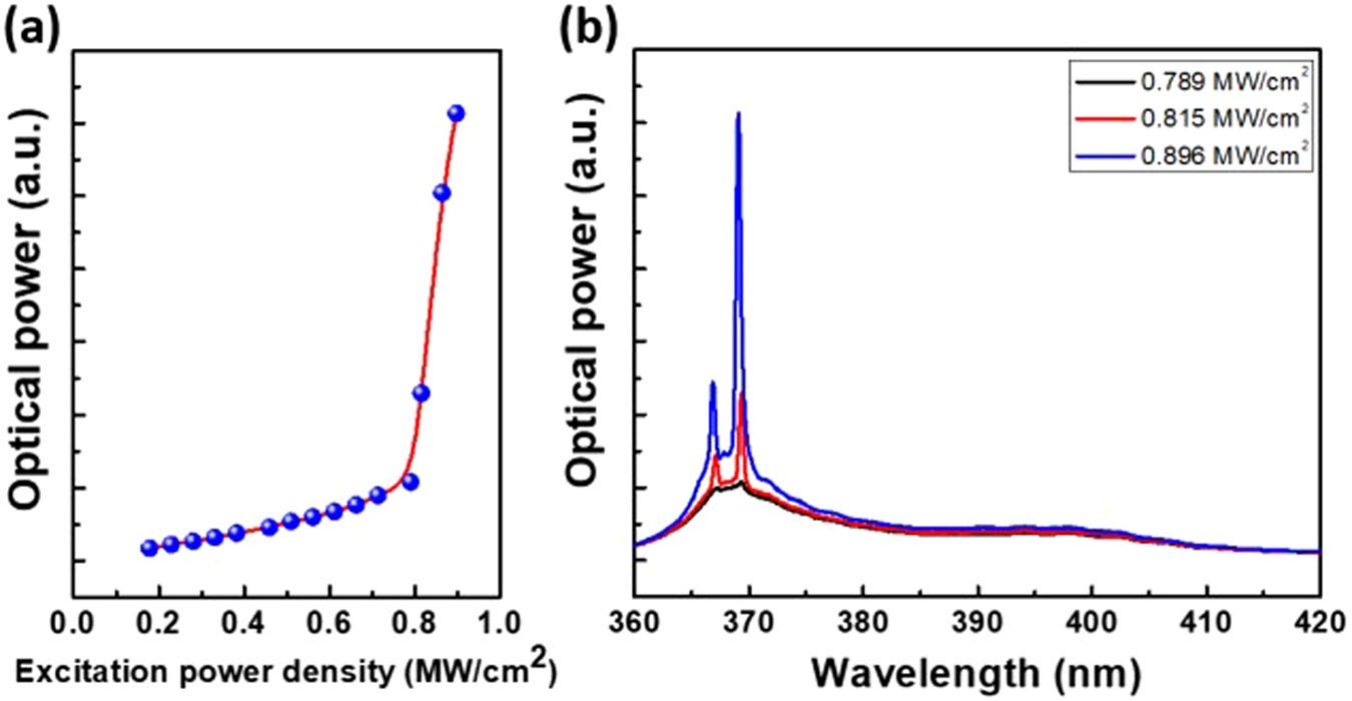
Room temperature lasing features of GaN MC with TiO_2_ HCG under non-polarized excitation. (**a**) Spectrally-integrated output power versus excitation power density. (**b**) Emission spectra at different excitation power density.

The TiO_2_ HCG MC was optically excited through the HCG by using a normally incident pulsed laser (Nd:YVO_4_) with a pulse length of 0.5 ns and a 1-kHz repetition rate at a wavelength of 355 nm. A 100× near UV infinity-corrected objective lens with a numerical aperture of 0.55 was used to focus the pumping beam to a diameter of 10 μm. The output emission was collected using a 600-μm core ultraviolet optical fiber and detected using a liquid-nitrogen-cooled charge-coupled device attached to a 320-mm single monochromator with a spectral resolution of 0.2 nm. A 360 nm filter was placed before the optical fiber to prevent signals from being influenced by the scattered pump laser. The optical output signals obtained at room temperature for the HCG MC lasers under nonpolarized excitation were measured, as shown in Fig. 3. The measured light in–light out curve unambiguously presents a lasing threshold at the excitation peak power density of 0.79 MW/cm^2^, as shown in Fig. 3(a). The laser emission spectrum shows two distinct peaks above the threshold, each with a linewidth of 0.5 nm. These two peaks are two different longitudinal modes inside the relatively long GaN cavity. Consider the longitudinal mode spacing equation: $L={\lambda }^{2}/2{n}_{g}\Delta \lambda$, where *L* is the effective cavity length, *n*_*g*_ is the group index of light inside the GaN cavity (wavelength-dependent refractive index of GaN used in the calculation was taken from refs^25,26^ and a large group index of 5.7 for 370 nm can be calculated because the wavelength is very close to the GaN resonant peak), and Δ*λ* is the measured mode spacing of 2.2 nm. With this equation, the effective cavity length was estimated to be approximately 5.4 μm, which is near the actual GaN thickness of 5.15 µm that was estimated from the SEM results.

**Figure 4:**
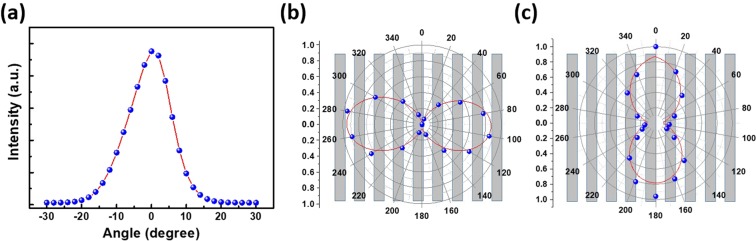
(**a**) Far-field divergence, (**b**,**c**) polar plots of normalized laser emission intensities for TM (period: 360 nm, DC: 38%) and TE (period: 375 nm, DC: 44%) polarization. The gray bars depict the grating direction of the HCG.

The far-field divergence and degree of polarization (DOP) of MC lasers operating above the threshold are depicted in Fig. 4. The far-field laser emission was collected using a fiber lens attached to a rotational stage. The lasing beam showed a full width at half maximum angle of 14°, as illustrated in Fig. 4(a). A polarizer was placed directly in front of the optical fiber to enable light output measurement as a function of the polarizer rotation angle. As displayed in Fig. 4(b,c), the DOP was calculated using (*I*_*max*_ − *I*_*min*_)/(*I*_*max*_ + *I*_*min*_), where *I*_*max*_ and *I*_*min*_ were the maximum and minimum relative light intensities, respectively. As shown in Fig. 4(b), the measured DOP of TM-polarized light was estimated to be 99.2%, which is proven to be the high linearly polarization perpendicular to the grating grooves. To verify that the polarization selection indeed stems from the grating, we further fabricated HCG MC lasers with different periods and DCs. We selected the period and DC of HCG to be 375 nm and 44%, respectively, so that the TE-polarized light would experience higher reflectance as calculated in Fig. 2. In the experiment, the TE-polarized (DOP: 75.6%) signal was expected to indicate a polarized laser output that is parallel to the HCG, which was in line with our simulation results. This clearly demonstrated that the lasing mode is determined by the grating design.

**Figure 5:**
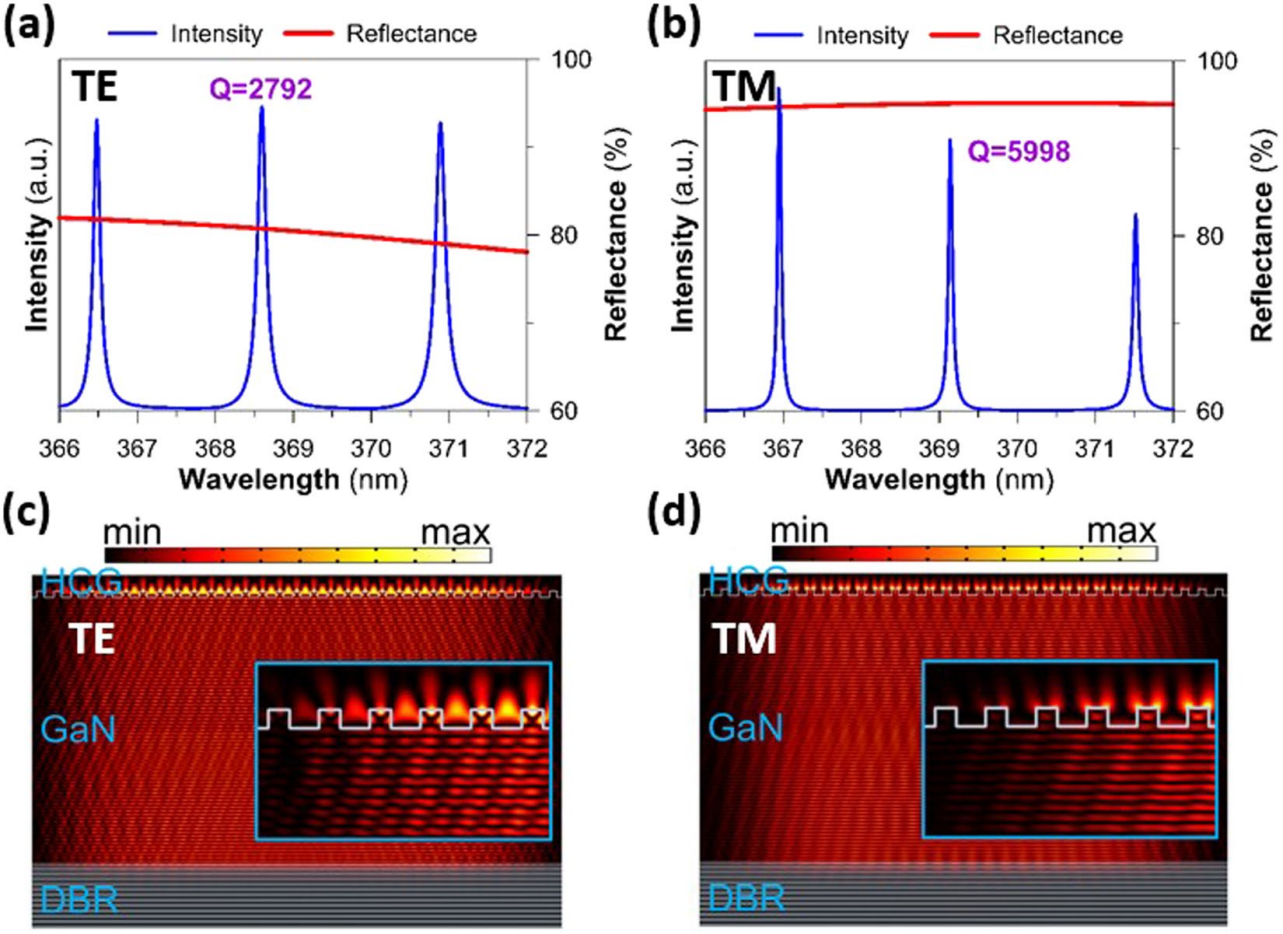
The calculated reflectance spectra (red lines) of the HCG and the emission spectra (blue lines) of the full cavity structure for (**a**) TE-polarized light and (**b**) TM-polarized light. Normalized electric field intensity distributions at resonance conditions of (**c**) the TE- and (**d**) TM-mode in the TiO_2_ HCG MC, with HCG h = 125 nm, Λ = 344 nm, and w/Λ = 42%. The resonant wavelengths are close to 369 nm. The insets are zoomed in images of TE- and TM-field intensity distribution near the edge of the simulation window.

Figure 5 presents simulated reflectance spectra of an individual HCG and the emission spectra of the full cavity structure for TE and TM modes. The optical simulations were obtained using the finite-element frequency domain technique by using COMSOL Multiphysics. To match the experimental condition, the thickness of GaN was set to 5.15 μm and the HCG parameters were as follows: *h* = 125 nm, *Λ* = 344 nm, and *w*/*Λ* = 42%. A wavelength-dependent refractive index was used for GaN and the DBR stack^25,26^ (assume that the refractive index of Ta_2_O_5_ is 2.279 and for SiO_2_ is 1.493). By considering the pump spot to be approximately 10 μm, the simulated domain contains a finite-size HCG MC laser with 31 grating bars of TiO_2_. Moreover, the full cavity structure is surrounded by a perfectly matched layer for avoiding the reflecting wave produced by the boundaries. The TE-polarization and TM-polarization reflectance spectra were obtained directly from the S-parameters and are illustrated in Fig. 5(a,b) (red lines) for a plane wave at normal incidence toward the GaN surface. As expected, a higher reflectance is observed for the TM-polarized light. The electrical field distributions are presented in Fig. 5(c,d). In addition, the field intensity distributions near the left-handed edge of the simulation window for TE and TM modes are shown in the insets of Fig. 5(c,d), respectively. It demonstrates the distribution of TE mode is slightly broader than that of TM mode which the electrical field distribution of the TE mode exhibits a larger lateral scattering. Thus, the optical loss of the TE mode was higher than that of the TM mode. This result implies that the designed and fabricated HCG reflector is good for a TM mode with higher reflectance. The output emission spectra (blue lines) were acquired by calculating the line average of the emitted electromagnetic energy by using a plane wave light source excited at a plane in the middle of the GaN layer. The mode spacing of approximately 2.19 nm agrees very closely with the experimental results. The cold cavity quality factor *Q* is as high as 5998 at a wavelength of 369.1 nm for the TM mode and is only 2792 at a wavelength of 368.6 nm for the TE mode, thus supporting the experimental finding of the dominant TM mode of the laser. From the experimental spectra, the *Q* value is estimated to be 738 (=369 nm/0.5 nm). The reasons for the deviation between the calculated and experimentally estimated *Q* values are optical absorption losses in the GaN bulk layers and the nonideal HCG (surface roughness, nonvertical sidewalls) in the real laser cavity. Note that the lasing threshold is probably lower than stated because approximately 73.5% of the pump intensity at a wavelength of 355 nm is reflected by the HCG for the TE-polarized light and 84.5% by the HCG for the TM-polarized light. This reflected intensity is not compensated while estimating the lasing threshold.

## Conclusion

GaN-based microcavity lasers with high reflectance TiO_2_ HCGs were successfully fabricated. The MC devices were optically pumped using a pulsed laser at a wavelength of 355 nm at room temperature. Moreover, the light in–light out curve exhibits an obvious threshold transition at a peak power density of 0.79 MW/cm^2^. The output laser emission was polarized perpendicularly to the HCG, as predicted by simulations. The divergence angle of the laser beam was 14°. Our demonstration of the GaN-based MC with a rigid HCG reflector not only proves the feasibility of using the robust and easily fabricated TiO_2_ HCG on the GaN surface but also sheds light on the benefits of HCGs in GaN-based light emitters operated in the wavelength range between green and ultraviolet regimes.

## References

[1] Higuchi Y, Omae K, Matsumura H, Mukai T (2008). Room-Temperature CW Lasing of a GaN-Based Vertical-Cavity Surface-Emitting Laser by Current Injection. Appl. Phys. Express..

[2] Lu TC, Kao CC, Kuo HC, Huang GS, Wang SC (2008). CW lasing of current injection blue GaN-based vertical cavity surface emitting laser. Appl. Phys. Lett..

[3] Takashima Y, Tanabe M, Haraguchi M, Naoi Y (2016). Highly polarized emission from a GaN-based ultraviolet light-emitting diode using a Si-subwavelength grating on a SiO2 underlayer. Opt. Commun..

[4] Lai YY, Chang TC, Li YC, Lu TC, Wang SC (2017). Electrically Pumped III-N Microcavity Light Emitters Incorporating an Oxide Confinement Aperture. Nanoscale Res. Lett..

[5] Chang TC (2017). High-temperature operation of GaN-based vertical-cavity surface-emitting lasers. Appl. Phys. Express..

[6] Bhattacharya A (2017). Room-Temperature Spin Polariton Diode Laser. Phys. Rev. Lett..

[7] Lu TC (2011). Room Temperature Current Injection Polariton Light Emitting Diode with a Hybrid Microcavity. Nano Letters..

[8] Huang MCY, Zhou Y, Chang-Hasnain CJ (2007). A surface-emitting laser incorporating a high-index-contrast subwavelength grating. Nat. Photonics..

[9] Huang MCY, Zhou Y, Chang-Hasnain CJ (2007). Nano electro-mechanical optoelectronic tunable VCSEL. Opt. Express..

[10] Huang MCY, Zhou Y, Chang-Hasnain CJ (2008). A nanoelectromechanical tunable laser. Nat. Photonics..

[11] Ansbaek T, Chung IS, Semenova ES, Yvind K (2013). 1060-nm Tunable Monolithic High Index Contrast Subwavelength Grating VCSEL. IEEE Photon. Tech. L..

[12] Chase C, Rao Y, Hofmann W, Chang-Hasnain CJ (2010). 1550 nm high contrast grating VCSEL. Opt. Express..

[13] Haglund E (2016). Demonstration of post-growth wavelength setting of VCSELs using high-contrast gratings. Opt. Express..

[14] Hofmann W (2010). Long-Wavelength High-Contrast Grating Vertical-Cavity Surface-Emitting Laser. IEEE Photon. J..

[15] Li K, Chase C, Qiao PF, Chang-Hasnain CJ (2017). Widely tunable 1060-nm VCSEL with high-contrast grating mirror. Opt. Express..

[16] Qiao PF, Li K, Cook KT, Chang-Hasnain CJ (2017). MEMS-tunable VCSELs using 2D high-contrast gratings. Opt. Lett..

[17] Kim J (2009). AlGaN membrane grating reflector. Appl. Phys. Lett..

[18] Lee J (2009). Polarization-dependent GaN surface grating reflector for short wavelength applications. Opt. Express..

[19] Wu TT (2012). Sub-wavelength GaN-based membrane high contrast grating reflectors. Opt. Express..

[20] Wang YJ (2014). Surface-normal emission from subwavelength GaN membrane grating. Opt. Express..

[21] Hashemi E (2015). TiO_2_ membrane high-contrast grating reflectors for vertical-cavity light-emitters in the visible wavelength regime. J. Vac. Sci. Technol. B..

[22] Lai YY (2015). Fabrication of SiC membrane HCG blue reflector using nanoimprint lithography. SPIE OPTO. International Society for Optics and Photonics..

[23] Gebski M (2015). High-contrast grating reflectors for 980 nm vertical-cavity surface-emitting lasers. In: High Contrast Metastructures IV. International Society for Optics and Photonics..

[24] Siefke T (2016). Materials pushing the application limits of wire grid polarizers further into the deep ultraviolet spectral range. Adv. Opt. Mater..

[25] Barker JAS, Ilegems M (1973). Infrared lattice vibrations and free-electron dispersion in GaN. Phys. Rev. B..

[26] Lin ME, Sverdlov BN, Strite S, Morkoc H, Drakin AE (1993). Refractive-Indexes of Wurtzite and Zincblende Gan. Electron. Lett..

